# The Plant Growth-Promoting Bacteria *Azospirillum amazonense*: Genomic Versatility and Phytohormone Pathway

**DOI:** 10.1155/2015/898592

**Published:** 2015-03-19

**Authors:** Ricardo Cecagno, Tiago Ebert Fritsch, Irene Silveira Schrank

**Affiliations:** ^1^Centro de Biotecnologia, Laboratório de Microrganismos Diazotróficos, Universidade Federal do Rio Grande do Sul (UFRGS), Porto Alegre, RS, Brazil; ^2^Departamento de Biologia Molecular e Biotecnologia, Instituto de Biociências, Universidade Federal do Rio Grande do Sul (UFRGS), CP 15005, 91501-970 Porto Alegre, RS, Brazil

## Abstract

The rhizosphere bacterium *Azospirillum amazonense* associates with plant roots to promote plant growth. Variation in replicon numbers and rearrangements is common among *Azospirillum* strains, and characterization of these naturally occurring differences can improve our understanding of genome evolution. We performed an *in silico* comparative genomic analysis to understand the genomic plasticity of *A. amazonense*. The number of *A. amazonense*-specific coding sequences was similar when compared with the six closely related bacteria regarding belonging or not to the *Azospirillum* genus. Our results suggest that the versatile gene repertoire found in *A. amazonense* genome could have been acquired from distantly related bacteria from horizontal transfer. Furthermore, the identification of coding sequence related to phytohormone production, such as flavin-monooxygenase and aldehyde oxidase, is likely to represent the tryptophan-dependent TAM pathway for auxin production in this bacterium. Moreover, the presence of the coding sequence for nitrilase indicates the presence of the alternative route that uses IAN as an intermediate for auxin synthesis, but it remains to be established whether the IAN pathway is the Trp-independent route. Future investigations are necessary to support the hypothesis that its genomic structure has evolved to meet the requirement for adaptation to the rhizosphere and interaction with host plants.

## 1. Introduction

The genus* Azospirillum* comprises free-living, nitrogen-fixing bacteria that are known as plant growth-promoting rhizobacteria (PGPR), which can colonize, by adhesion, the root surface or the intercellular spaces of the host plant roots. The potential role of the PGPR in association with economically important cereals and other grasses is to promote plant growth by several mechanisms including nitrogen fixation and phytohormone production [1]. Several species of* Azospirillum* are able to secrete phytohormones such as auxins, gibberellins, cytokinins, and nitric oxide as signals of plant growth promotion [2, 3].

*Azospirillum* genomes, as previously suggested for various strains, are larger and are comprised of multiple replicons indicating a potential for genome plasticity [4]. Genomic rearrangements can occur spontaneously where replicons can be lost upon the formation of new megaplasmids [5, 6]. Moreover, genome sequencing of some* Azospirillum* species revealed that significant part of the genome has been horizontally acquired [6]. Up until now, 16* Azospirillum* species have been characterized; however complete genomic sequences of only* Azospirillum brasilense*,* Azospirillum lipoferum*,* Azospirillum* sp. B510, and a draft of* Azospirillum amazonense* genome have been published [7].

*Azospirillum amazonense* was found to be associated with the roots and rhizosphere of several grasses including sugar-cane, maize, sorghum, and rice revealing a broad ecological distribution in Brazil. Studies revealed that* A. amazonense* is phylogenetically closer to* Rhodospirillum centenum* and* Azospirillum irakense* than to* A. brasilense*. Unlike other* Azospirillum* strains,* A. amazonense* can grow in the presence of sucrose as sole carbon source and is also better adapted to soil acidity, which offers the bacterium additional advantages for colonization of plant root tissue in acid environments [8, 9]. Moreover,* A. amazonense* genomic analyses revealed the presence of genes not commonly distributed in other* Azospirillum* species such as those responsible for the utilization of salicin as carbon source (similar to* A. irakense*) and a gene cluster (RubisCO) implicated in carbon fixation (*A. lipoferum* is able to grow autotrophically by means of RubisCO, but the presence of the genes has not yet been demonstrated) [7]. However, our understanding of phytohormone production in* A. amazonense* is still incomplete.

The genomic plasticity of* A. amazonense* is probably related to the versatile gene repertoire present in the genome of this bacterium suggesting that horizontal gene transfer may have an impact on the adaptation and evolution of this species. Gene organization and phylogenetic analysis demonstrated that genes coding for proteins responsible for the nitrogen fixation process, carbon fixation (RubisCOs), and molecular hydrogen oxidation (hydrogenases) is more closely related to Rhizobiales members than to related species [7].

To further examine the importance of* A. amazonense* genetic variability, an* in silico* comparative genomic analysis using subtractive hybridization was performed using total coding sequences (CDS) from* A. amazonense* to compare with genomes of closely related bacteria. The analysis of conserved and specific* A. amazonense* coding sequences indicated features that distinguished* A. amazonense* from other* Azospirillum* species. Furthermore, the specific interesting features related to phytohormone production may provide several cues to establish* A. amazonense* pathways for auxin biosynthesis.

## 2. Material and Methods

### 2.1. Bacteria Selection and Genome Access

We have previously generated a good quality draft genome sequence of the* A. amazonense* Y2 (ATCC 35120) strain [7]. In this paper, the draft genome sequences were annotated and analyzed for the presence of specific regions, and during the BLAST search best-hits were detected with different bacteria, such as* Rhodospirillum*,* Azospirillum*,* Bradyrhizobium*, and* Caulobacter*.

Therefore, the* A. amazonense* comparative genomic analyses were performed using bacterial genomes including six species for which publicly closed genomes were available (Table 1). All genomes were downloaded from NCBI on January 10, 2013. The accession numbers used in this study are* Azospirillum amazonense* Y2 PRJNA73583, PRJNA65263;* Azospirillum* sp. B510 projects PRJNA46085, PRJDA32551;* Azospirillum brasilense* Sp245 PRJEA162161, PRJEA70627;* Azospirillum lipoferum* 4B PRJNA82343, PRJEA50367;* Rhodospirillum centenum* SW project PRJNA58805;* Bradyrhizobium japonicum* USDA 110 projects PRJNA57599 and PRJNA17; and* Caulobacter segnis* ATCC 21756 project PRJNA41709.

### 2.2. Annotation and Subtractive Hybridization

Reannotation of* A. amazonense* protein-coding genes was performed with a following procedure, which consists of two phases: initially the* A. amazonense* contigs were compared with the* Azospirillum* sp. B510 genome followed by functional annotation of each coding sequence (CDS) based on comparison with known sequences of the other six selected genomes using the Xbase Annotation Service [10]. All coding sequences predictions were manually checked for conservation in case of multiple hits, and only the alignments with best-hit results were selected from each genome. Information related to Cluster of Orthologous Group and KEGG pathway was added to the annotation using the server for metagenomic analysis (WebMGA) [11]. Annotation was based on comparison to protein clusters and on the BLAST results.

The subtractive hybridization using the mGenomeSubtractor program [12] was applied to run BLAST searches of the* A. amazonense* genome against multiple bacterial genomes for* in silico* comparative genomic analyses in order to characterize the unique sequences of* A. amazonense*. Proteins possibly related to phytohormones were analyzed in the Arabidopsis Hormone Database (AHD) [13], and proteins with homology (*H*) values more than 0.1 were arbitrarily defined as conserved coding sequences.

## 3. Results and Discussion

### 3.1. Comparative Analyses and Specific Protein Coding Sequences

The draft genome sequence of* A. amazonense* consists of 7,044,835 bp with 3,319 predicted coding sequences (CDS) where 2,299 have similarity with genes with known functions and 1,020 codes for hypothetical proteins or proteins of unknown function [7]. Although the estimated coverage of the genome was 35x, the number of predicted coding sequences was lower when compared with the other species of* Azospirillum* where the total number of coding sequences ranges from 6,093 to 7,557 (Table 1).

In order to clarify the genomic coding sequences content of* A. amazonense*, two alternative comparative approaches using the Xbase Annotation Service were performed. Initially, to assess the coverage of the predicted gene repertoires a BLAST search was performed with only the* Azospirillum* sp. B510 genome. The total number of predicted protein-coding genes was 5,496 of which 2,165 were annotated as proteins of unknown function or hypothetical proteins. These numbers are similar to what is found in* A. lipoferum* and* Azospirillum* sp. B510 (Table 1). These results including the 5,496 sequences can be accessed using the http://www.xbase.ac.uk/annotation/results/rWn50Rn6LVRucf55SWfsvHfoqdpmd655/.

The second approach used an* A. amazonense*-vs-all (six selected genomes) BLAST to examine the overall similarity of the* A. amazonense* genome with closely related bacteria, and the results are shown in Table 2. The whole-genome comparisons revealed that the number of coding sequences found to be conserved and characterized as best-hits varied in each bacterium, from 3,126 (present in* Azospirillum* sp. B510) and 1,508 (present in* Rhodospirillum centenum*) to 2,846 (present in* R. centenum*) and 440 proteins (present in* A. lipoferum*), respectively. It is important to point out that the majority of the orthologs showing best-hit results were found with the* R. centenum* genome supporting previous suggestions of a close evolutionary relationship between* A. amazonense* and* R. centenum* [7, 14]. Interestingly, the number of coding sequences with best-hits orthologs found in the other* Azospirillum* species is almost equivalent to those found in the genome of bacteria from other genera, such as* Bradyrhizobium japonicum* and* Caulobacter segnis* (Table 2).

These unexpected results may support previous reports related with the genome repertoire of* A. amazonense* where horizontal gene transfer may be one of several events that result in an intragenera genomic plasticity. Phylogenetic analysis indicated the close relationship of the* A. amazonense* enzymes encoded by the gene cluster related to carbon fixation (RubisCo) and by genes related to nitrogen fixation (*nif*) processes with those from some species of the order Rhizobiales. Moreover, some features, such as the genetic organization of the carbon-fixation cluster and of the* nif* cluster of* A. amazonense,* are similar to the homolog cluster of the* Bradyrhizobium* species [7].

In conclusion, from the total 5,496 CDS found in the* A. amazonense* genome approximately half of the coding sequences have an ortholog in other closely related bacteria. However, using this methodology's numbers varying from 2,370 to 2,650 CDS showed lower degrees of similarity (*E* value > 10^−10^) with coding sequences present in the genome of the compared bacteria.

**Figure 1:**
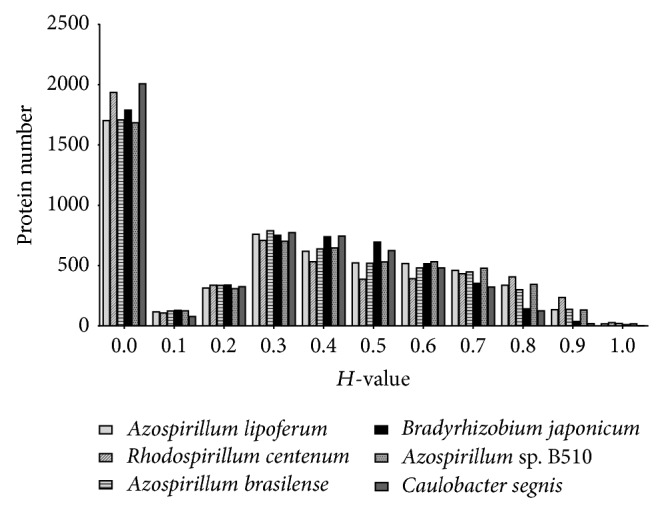
Histogram distribution of predicted proteins in* A. amazonense* compared with six closely related genomes using BLASTP-based homology value (*H* value). The *H*-value reflects the degree of similarity in terms of length of match and the degree of identity at amino acid level between the matching CDS in the subject genome and the query CDS examined with *E* value > 10^−8^.

Comparative genomic analysis using* in silico* subtractive hybridization allowed searching for specific proteins of the* A. amazonense* genome against multiple closely related bacterial genomes. Therefore, to determine the possible differences between the* A. amazonense* genome and each of the selected six closely related genomes, an* in silico* subtractive hybridization technique was applied. The histogram of *H*-values (Figure 1) was used to set the cutoff to discriminate between* A. amazonense*-specific and conserved coding sequences. Proteins with homology (*H*) values of less than 0.42 and more than 0.64 were arbitrarily defined as specific and conserved coding sequences, respectively [12]. This cutoff value was proposed by Shao et al. [12] and has been used in comparative genomic analyses to differentiate strains of pseudomonads [12, 15] or to compare genomes of species from the genus* Erwinia* [16].

The subtractive hybridization approach revealed different profiles in gene number of specific and conserved proteins for the* A. amazonense* genome against the six others (Table 3). The number of proteins found to be conserved varied in each bacterium, from 948 CDS in* A. lipoferum* to 317 CDS in* B. japonicum*. Interestingly, the number of* A. amazonense*-specific proteins was similar when compared with the six bacteria varying from 4,043 (*C. segnis*) to 3,571 (*Azospirillum *sp. B510). Moreover, the specific proteins vary among the* Azospirillum* genomes analyzed from 3,571 to 3,689 only, indicating that these coding sequences are unique to the* A. amazonense* genome. Analyses of Figure 1 show that the majority of specific proteins in* A. amazonense* have *H*-values less than 0.1, suggesting that* Azospirillum* species are evolutionally diverse. This is consistent with previous studies that had proposed that some regions of the genome of* Azospirillum* species were acquired from distantly related bacteria from horizontal transfer [6].

To further characterize the global profile of* A. amazonense*-specific coding sequences, an* in silico* subtractive hybridization comparative analysis was performed with total* A. amazonense* putative coding sequences versus all six genomes, simultaneously. A total of 142 conserved CDS and 2,483 specific CDS were identified (see Supplementary Table 1 in Supplementary Material available online at http://dx.doi.org/10.1155/2014/898592). For function classification and pathway assignment, all specific and conserved* A. amazonense* coding sequences were classified to 20 different functional classes based on Clusters of Orthologous Groups (COG) (Table 4). The comparison of the* A. amazonense* genome and the other six available closely related genomes with regard to the functional category revealed that 1,196 specific CDS from* A. amazonense* were distributed among the different classes of orthologous clusters and that 1,287 specific CDS were unclassified being considered hypothetical products.

Detailed analysis of the* A. amazonense*-specific CDS from Table 4 (and Supplementary Table 1) indicates special attention to the coding sequences classified in the Signal Transduction Mechanisms and Secondary Metabolites Biosynthesis, Transport and Catabolism functional class. Among the 137 CDS classified in the Signal Transduction Mechanisms functional category, there were protein coding sequences similar to cytokinins (ZP_08868173.1, ZP_08868457.1) and ethylene response (ZP_08867667.1) that could be related to phytohormone production (Supplementary Table 1). In particular, we have paid attention to a coding sequence related to lysine/ornithine N-monooxygenase (ZP_08869952.1) similar to flavin-containing monooxygenase from* Arabidopsis thaliana* (YUCCA9) involved in auxin synthesis in plants [17, 18] found in the Secondary Metabolites Biosynthesis, Transport and Catabolism functional class (Supplementary Table 1). Therefore, to better understand the auxin biosynthesis pathways in* A. amazonense*, studies attempting to define coding sequence related to phytohormone production were performed.

### 3.2. Phytohormone Production Related Sequences

The improvement of plant growth upon* Azospirillum* inoculation is attributed, as one of many factors, to the production of auxin by these bacteria [19]. Indole-3-acetic acid (IAA) is considered the most important auxin implicated in different aspects of plant growth. In bacteria, the two most common routes for indole-3-acetic acid biosynthesis are the IAM (indole-3-acetamide) and the IPyA (indole-3-pyruvate) pathways [20]. However, in* A. brasilense*, besides these two tryptophan-dependent pathways, an additional tryptophan-independent pathway was identified [21].

Similar to other* Azospirillum* species,* A. amazonense*, as typical plant-growth promoting rhizobacteria, stimulate root proliferation [22, 23]. However, genes responsible for biosynthesis and secretion of phytohormones are poorly described in this species. Previous works on* A. amazonense* genome sequence and annotation were unable to localize genes related to the IAM or IPyA pathways (*iaaM*,* iaaH*, and* ipdC*) and were able to identify only the presence of a coding sequence similar to nitrilases responsible for the conversion of indole 3-acetonitrile (IAN) to IAA in plants [7]. Therefore, the presence of coding sequences homologous to nitrilase and to flavin-containing monooxygenase (this paper) suggests that* A. amazonense* could use alternative pathways closely related to those found in plants.

**Figure 2:**
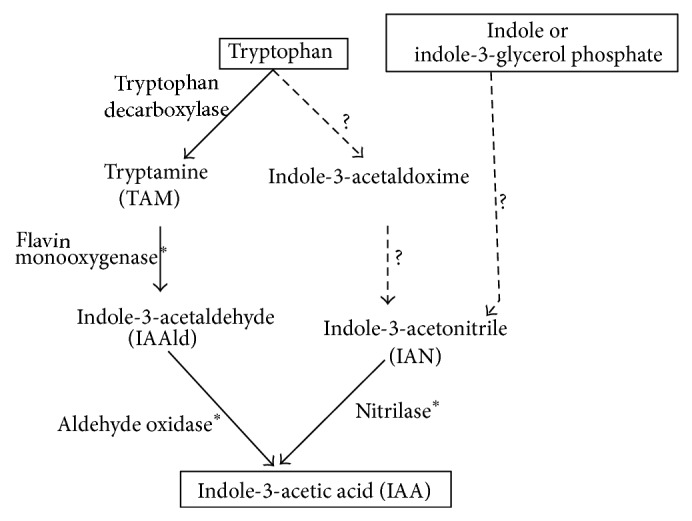
*A. amazonense* pathways of IAA biosynthesis. Tryptophan-dependent pathways or tryptophan-independent pathways (starting from indole or indole-3-glycerol phosphate) are indicated based on routes found in plants and bacteria. Enzymes indicated with an asterisk have been identified in* A. amazonense*, and routes indicated as dotted lines indicated that the precursor of IAN may or may not be tryptophan.

Biosynthetic pathways for IAA have been fully investigated and tryptophan-dependent and Trp-independent routes have been studied [20, 24, 25]. Although genes coding for proteins related to the bacterial common routes IAM (indole-3-acetamide route) and IPyA (indole-3-pyruvic route) was not found in the* A. amazonense* genome, the identification of flavin-monooxygenase and nitrilase enzymes suggests the presence of the TAM (tryptamine route) and IAN (indole-3-acetamide route) pathways for IAA synthesis in this bacterium (Figure 2). It is well known that nitrilases in plants (maize and* Arabidopsis thaliana*) and also in* Bacillus amyloliquefaciens* were shown to hydrolyze indole-3-acetonitrile (IAN) to IAA [25, 26]. Moreover, evidence for the IAN and TAM pathways has been reported in* A. brasilense* [21].

Aiming to unveil the IAA pathways in* A. amazonense,* a search for other enzymes involving the TAM pathway was performed. The genome of* A. amazonense* contains an aldehyde oxidase-coding sequence (WP_004273557) homolog (query cover 91%; 33% identity; *E* value *e* − 99) to the* A. thaliana* AAO1 gene (AED92912) that is capable of oxidizing indole-3-acetaldehyde to indole-3-acetic acid with high efficiency [27]. Therefore, oxidation of indole-3-acetaldehyde by* A. amazonense* aldehyde oxidase is likely to represent the TAM route transforming indole-3-acetaldehyde (IAAld) to produce IAA phytohormone in this bacterium (Figure 2). To conclude,* A. amazonense* appears to possess only one regulated Trp-dependent route for IAA synthesis, the TAM pathway, while* A. brasilense* possesses two differently regulated routes, namely, the IPyA and the TAM pathways [21]. Furthermore, the alternative route that uses IAN as an intermediate, the IAN pathway, appears to be present in both species, but it remains to be established whether the IAN pathway is the Trp-independent route.

To further understand the phytohormone biosynthesis pathway in* A. amazonense,* an* in silico* comparative analysis was performed with total coding sequences from* A. amazonense* versus all proteins deposited in the Arabidopsis Hormone Database. Furthermore, the comparative analysis was also performed with coding sequences in the auxin response transcriptome data of* A. brasilense* [28]. A total of 54* A. amazonense* CDS revealed similarity with proteins related to hormone production in plants and bacterial auxin signal transduction pathways (Supplementary Table 1). The presence of these coding sequences suggests that IAA could be a signal that alters gene expression in* A. amazonense* similar to that found in* A. brasilense* [28]. Moreover, the genome of* A. amazonense* has coding sequences that could be related to other hormone pathways similar to those described for other* Azospirillum* species [19, 21].

Another beneficial effect provided by the association of soil bacteria with plants could be due to the plant hormone ethylene, which can inhibit plant growth by regulating several developmental aspects [29]. Similar to other plant growth-promoting rhizobacteria, a coding sequence homologous to 1-aminocyclopropane-1-carboxylate (ACC) deaminase (WP_004272971.1) has been identified in the* A. amazonense* genome. Previous reports have suggested that* A. amazonense* can stimulate plant growth by producing or metabolizing plant hormones and the presence of ACC deaminase which can hydrolyze ACC, the immediate precursor of the plant hormone ethylene, could be involved by lowering the plant ethylene levels and increasing plant growth. Moreover, the identification of the octaprenyl diphosphate synthase enzyme (ZP_08868744) could be related to cytokinin biosynthesis by a known hormone that affects plant growth and yield. Although the auxin hormone is considered a major class of hormones regulating plant growth, cytokinins or ethylene-related phytohormones could interact with auxin leading to root system development.

In conclusion, it appears that the rhizosphere bacterium* A. amazonense* is able to produce IAA through the tryptamine and indole-3-acetonitrile pathways and similar to* A. brasilense* could alter gene expression in response to the presence of auxin. Moreover, the role as plant-growth-promoting bacteria could be related to IAA production or to its ability to metabolize the ethylene precursor (ACC) and thereby increases the growth of the root system. Furthermore, the multiple genome comparison performed with* A. amazonense* and closely related bacteria supports previous evidence concerning* A. amazonense* genomic versatility and that several genes could have been acquired from distantly related bacteria [6]. Future investigation of* A. amazonense* is necessary to support the hypothesis that its genomic structures have evolved to meet the requirements for adaptation to the rhizosphere and interaction with host plants.

## Supplementary Material

Supplementary Table: Annotation and characterization of *A. amazonense* proteins sequences in curated databases.
List of proteins identified in *A. amazonense* genome by automatic prediction using the *Xbase Annotation Service* (this work) and previously deposited in GenBank (Amz access). This proteins were classified using curated databases: AHD - Arabidopsis Hormone Database, CDD - Conserved Domain Database, COG - Cluster of Orthologous Groups, KEGG - Kyoto Encyclopedia of Genes and Genomes, PRK - Protein Clusters Database, PFAM - Protein Families Database, TIGR - The Institute for Genomic Research by J. Craig Venter Institute, GO - Gene Ontology, E. C. Number - Enzyme Commission Number, VFDB - Virulence Factors Database, DEG - Database of Essential Genes, KOG - Eukaryotic Orthologous Groups and Fasta title - fast format protein identification

## Figures and Tables

**Table 1:** General features for the bacteria genomes used in the comparative analysis.

Bacteria	Genome size	Total number of CDS	Assembly reference number
*Azospirillum amazonense *	7,044,835	3,319^*^	ASM22599v1
*Azospirillum brasilense *	7,530,241	7,557	ASM23736v1
*Azospirillum lipoferum *	6,846,400	6,093	ASM28365v1
*Azospirillum *sp. B510	7,599,738	6,309	ASM1072v1
*Rhodospirillum centenum *	4,355,543	4,003	ASM1618v1
*Caulobacter segnis *	4,655,622	4,139	ASM9228v1
*Bradyrhizobium japonicum *	9,105,828	8,317	ASM1136v1

^*^The total number of *A. amazonense* CDS was published by Sant'Anna et al. 2011 [7].

**Table 2:** Predicted distribution of coding sequences (CDS) in *A. amazonense* draft genome and in the complete genome of other bacteria.

Comparisons	Conserved CDS		Specific CDS
	Best-hits	Total number	
A. a. versus *Azospirillum brasilense *	583	3,031	2,465
A. a. versus *Azospirillum lipoferum *	440	3,084	2,412
A. a. versus *Azospirillum *sp. B510	533	3,126	2,370
A. a. versus *Rhodospirillum centenum *	1,508	2,846	2,650
A. a. versus *Caulobacter segnis *	711	2,852	2,644
A. a. versus *Bradyrhizobium japonicum *	632	2,970	2,526

A. a.: *Azospirillum amazonense*.

Protein coding sequences with *E* value >10-10 were considered specific CDS (using the Xbase Annotation Service).

**Table 3:** Numbers of specific proteins for *A. amazonense* genome against six closely related genomes.

Comparisons	*A. amazonense *		
	Specific CDS	Conserved CDS	Other CDS
*Azospirillum brasilense *	3,689	948	859
*Azospirillum lipoferum *	3,606	746	1,144
*Azospirillum *sp. B510	3,571	793	1,132
*Rhodospirillum centenum *	3,697	770	1,029
*Caulobacter segnis *	4,043	382	1,071
*Bradyrhizobium japonicum *	3,880	317	1,299

The *in silico* subtractive hybridization analysis was performed with *A. amazonense* total coding sequences (CDS) against the proteins from the six genomes.

Proteins with homology (*H*) value less than 0.42 and more than 0.64 were arbitrarily defined as specific and conserved CDS, respectively, and other CDS were defined with *H* values between 0.42 and 0.64.

**Table 4:** Protein categories encoded by *A. amazonense* specific and conserved genes identified by *in silico* subtractive hybridization.

CDS assigned function^*^	Specific CDS	Conserved CDS
Transcription	143	11
Signal transduction mechanisms	137	5
Inorganic ion transport and metabolism	111	1
Carbohydrate transport and metabolism	101	4
Cell wall/membrane/envelope biogenesis	94	1
Amino acid transport and metabolism	86	19
Energy production and conversion	49	27
Secondary metabolites biosynthesis, transport, and catabolism	47	3
Cell motility	44	0
Coenzyme transport and metabolism	41	4
Lipid transport and metabolism	41	10
Intracellular trafficking, secretion, and vesicular transport	37	1
Defense mechanisms	34	0
Replication, recombination, and repair	33	3
Posttranslational modification, protein turnover, and chaperones	27	16
Translation, ribosomal structure, and biogenesis	14	27
Nucleotide transport and metabolism	12	12
Cell cycle control, cell division, and chromosome partitioning	4	1
RNA processing and modification	2	0
General function prediction only or function unknown	352	11

Proteins with homology (*H*) value less than 0.42 and more than 0.64 were arbitrarily defined as specific and conserved CDS, respectively.

^*^CDS assigned function was based on the COGs according to BLAST search.
